# High myopia and its risks

**Published:** 2019-05-13

**Authors:** Katie Williams, Christopher Hammond

**Affiliations:** 1 Ophthalmology Specialist Trainee Year 6: King's College London, UK.; 2 Frost Professor of Ophthalmology: King's College London, UK.

**High myopia increases the risk of blinding eye conditions, so regular follow-up is essential.**

**Figure:**
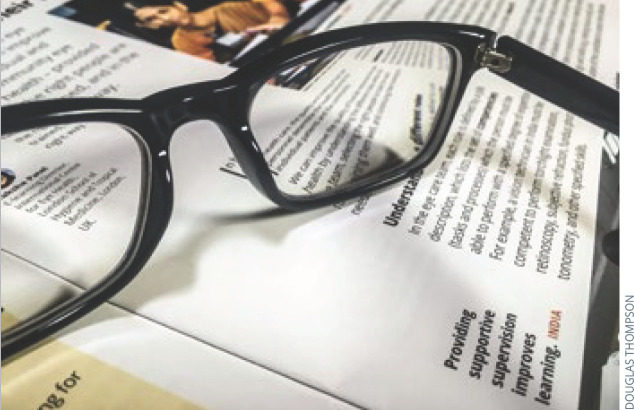
High myopia increases the risk of potentially blinding eye conditions. UK

High myopia is said to occur when a person's myopia progresses until they need −5 dioptres (D) or more of spherical correction,^1^,^2^ although the definitions used to grade myopia are variable.

The prevalence of myopia is increasing globally.^3^ It has been predicted that, by the year 2050, high myopia will affect 9.8% of the global population; a total of 938 million people.^4^ The highest prevalence of myopia is seen in younger adults, particularly in urbanised East and Southeast Asian countries.^2^

**High myopia**

The definition of high myopia as ≤ −5 D was adopted as the World Health Organization (WHO) definition in 2015. A person who needs ≤ −5 D of correction has a visual acuity that is far worse than the threshold for blindness (–3/6 in the better eye).

Even when appropriate refractive correction is provided, myopia continues to place an individual at an increased risk of sight-threatening diseases, including^5^,^6^:

- Glaucoma (open-angle)
- Cataract (nuclear, cortical and posterior subcapsular)
- Retinal tears which may lead to a retinal detachment
- Myopic maculopathy or myopic macular degeneration

The incidence of these conditions is greatest in individuals with high myopia.

**Glaucoma.** A systematic review of the available evidence concluded that the risk of developing glaucoma was nearly 50% higher (or one and a half times as high) in individuals with moderate to high myopia, compared to those with low myopia (odds ratios [OR] of 2.5 and 1.7 respectively).^7^

**Cataract**. Higher rates of cataract surgery are seen in individuals with high myopia. Based on the available evidence, they are 17% *more* likely than those with moderate myopia to need cataract surgery (odds ratios of 3.4 and 2.9, respectively).^8^

**Retinal detachment.** The risk of developing a retinal detachment is five or six times greater in people with high myopia (OR >20) compared to those with low myopia (OR <4).^9^ People with high myopia have longer eyes (axial elongation), which means that the retina is more stretched and therefore prone to peripheral retinal tears. In addition, myopic eyes have a degenerate vitreous that is more likely to collapse and separate from the retina, also increasing the risk of retinal tears. High myopia can also cause central retinal degenerative changes such as posterior staphyloma, lacquer cracks and chorioretinal atrophy; these have been used to grade myopic maculopathy.^10^

**Odds ratios (OR)** are used to express relative risk in case-control studies such as those referred to in this article. In these studies, participants are grouped according to the outcome, e.g., whether they had cataract surgery or not, and then information is obtained about their exposure to a risk factor. In these studies, the risk factor is high myopia.

**Myopic macular degeneration (maculopathy).** The risk of macular degeneration due to myopia rises sharply with age and increasing myopia.^9^ Myopic maculopathy may take the form of atrophic changes or be complicated by choroidal neovascular membrane (CNV) formation.^10^ Advanced myopic maculopathy causes loss of central vision and there is currently no treatment for the atrophic form. With the increasing prevalence of myopia, visual impairment caused by this condition will continue to rise.

## Speaking to patients with myopia

It is important to make patients aware of these potentially sight-threatening conditions and that their risk appears to be proportionate their degree of myopia. Any sight loss should therefore prompt patients to seek a complete ophthalmic assessment.

Retinal detachment can affect any age group. Tell patients to contact an eye specialist immediately if they see flashing lights (usually seen in dim light in the temporal peripheral field) or floaters, or if they experience visual field loss. They must undergo an urgent dilated exam to exclude retinal tears and/or detachment.

Central visual loss as a result of advanced myopic macular degeneration can affect people of working age, so examine the macula at every visit. Individuals who develop CNV may be treated with intravitreal anti-VEGF therapies.^11^ Refer those with central visual loss for low vision assessment and/or offer hand-held magnifiers.

Because the risk of open-angle glaucoma increases in individuals with high myopia, it is wise to assess intraocular pressure and optic disc appearance at every visit. Assess visual fields if possible.

Ophthalmic workers should acknowledge high myopia as a significant cause of visual impairment and a risk factor for a number of sight-threatening conditions.

**Key messages**

- High myopia is becoming more common
- Even if the refractive error is corrected, the eye is at risk of visual impairment, particularly if the myopia is ≤ −5 D
- Myopia increases the risk of open-angle glaucoma, retinal detachment, and myopic macular degeneration
